# A Letter from Dr. Hayward

**Published:** 1842-10-22

**Authors:** Geo. Hayward

**Affiliations:** Boston


### A LETTER FROM DR. HAYWARD.

To the Editors of the Medical Examiner.

GENTLEMEN—I regret that you did not publish the note which I addressed to you some time since in relation to Dr. Clymer's paper on the mortality after amputations. I should not, however, have called your attention to the subject again, were it not for the statement that appeared in the Examiner of the 8th instant, respecting a communication from that gentleman.

Though this is a very unsatisfactory explanation, for he apparently does not think an *apology* necessary, I should have passed it by in silence, if he had not fallen into another error in attempting to correct me. In the last sentence of the statement referred to, it is said, " Dr. Hayward's report, correctly stated, was 67 amputations, of whom 15 died, and not 70, the number given by Dr. Hayward in the extract from his note which we published last week." This is not correct. The number of amputations was 70, precisely as I stated, which were performed on 67 patients.

Thus it appears, that in the first notice of my paper in the statistics of amputations performed at the Massachusetts General Hospital, the writer makes three gross errors in two short lines; and then in what is intended for an explanation, he deliberately charges me with making a false statement in the note which I addressed to you on the subject; a charge which he would have seen was wholly groundless, if he had taken the trouble to read the article, of which he pretended to give an account, and which he can find in the 26th vol. of the American Journal of Medical Sciences.

I trust that you will do me the justice to publish *this* note in your Journal.

Respectfully, yours, GEO. HAYWARD.

*Boston, October* 15*th*, 1842.

